# Potential Diagnostic and Therapeutic Uses of DPT in Acute Type A Aortic Dissection

**DOI:** 10.1155/cdr/8896404

**Published:** 2026-02-06

**Authors:** Ting Wei, Xiaopeng Yang, Chao Shi

**Affiliations:** ^1^ Department of Cardiovascular Surgery, The First Affiliated Hospital of Bengbu Medical University, Bengbu, China, bbmc.edu.cn

**Keywords:** acute Type A aortic dissection, bioinformatics analysis, DPT, PPI, WGCNA

## Abstract

**Purpose:**

Acute Type A aortic dissection (ATAAD) is a catastrophic cardiovascular emergency with high mortality and few treatment options. Diagnostic biomarkers or targeted treatments remain in the rudimentary stage, complicating early detection and intervention. The aim is to discover novel diagnostic and therapeutic biomarkers for ATAAD through integrated bioinformatics and experimental validation.

**Methods:**

Differentially expressed genes (DEGs) were identified using the “limma” package in R, applying the combined, normalized, and batch‐effect‐corrected microarray datasets GSE52093 and GSE98770. Functional enrichment analyses (GO and KEGG), protein–protein interaction (PPI) network construction, and weighted gene coexpression network analysis (WGCNA) were performed to identify key genes. Key genes were validated by qPCR, immunofluorescence, and functional assays in human aortic smooth muscle cells (HASMCs) and an independent dataset (GSE153434).

**Results:**

There were 441 DEGs with 164 upregulated and 277 downregulated genes. These hub genes also overlapped with four key genes (DPT, ITGA5, HGF, and PLAUR) in the key WGCNA module. Of these, DPT was downregulated compared with ATAAD tissues. DPT knockdown induced HASMC migration and inhibited HASMC proliferation, as assessed by functional assays. The diagnostic potential of these genes, especially of DPT, was confirmed using ROC analysis.

**Conclusion:**

DPT is a promising diagnostic and therapeutic biomarker for ATAAD. Downregulation may also disturb extracellular matrix homeostasis and smooth muscle cell function, leading to aortic wall instability. These findings provide a foundation for future research on DPT‐targeted interventions for ATAAD.

## 1. Introduction

Acute Type A aortic dissection (ATAAD) results from an aortic intimal tear that leads to the entry of blood into the aortic wall, separating its layers and leading to the formation of a false lumen and ascending aortic involvement within 14 days [1, 2]. Aortic dissection (AD) is a severe surgical emergency, and when untreated, the condition is associated with mortality rates as high as 33% and 50% within the first 24 and 48 h [3]. ATAAD currently necessitates immediate surgery upon diagnosis [4], and the operation itself is very challenging, such that it poses a significant test of the attending surgeon’s skills [5–7]. Technical, economic, and geographical factors all shape the management of this disease [3]. As the early symptoms of ATAAD often overlap with those of other conditions, delayed diagnosis is common, limiting the window for effective treatment and leading to higher mortality [7]. No specific diagnostic biomarkers or therapeutic agents are currently available for ATAAD, underscoring the need to define more robust early diagnostic markers for this condition and to develop treatment strategies to reduce patient mortality.

Gene microarrays are often used to compare gene expression patterns between standard and disease‐related samples, and the Gene Expression Omnibus (GEO) database provides a wealth of publicly available microarray data [8]. Human aortic smooth muscle cells (HASMCs) are the primary cells that comprise the aortic media and help maintain aortic functional and structural integrity. These HASMCs are associated with arterial remodeling, which underlies the pathology of aortic AD, and were thus the primary cell type of interest in this study. Transcriptomic microarray data from the GEO database associated with ATAAD were analyzed to identify differentially expressed genes (DEGs) between standard and ATAAD samples. These DEGs were subjected to Gene Ontology (GO) and Kyoto Encyclopedia of Genes and Genomes (KEGG) pathway enrichment analyses. At the same time, hub genes were identified by establishing a protein–protein interaction (PPI) network. A weighted gene coexpression network analysis (WGCNA) was also performed on the training dataset.

**Figure 1:**
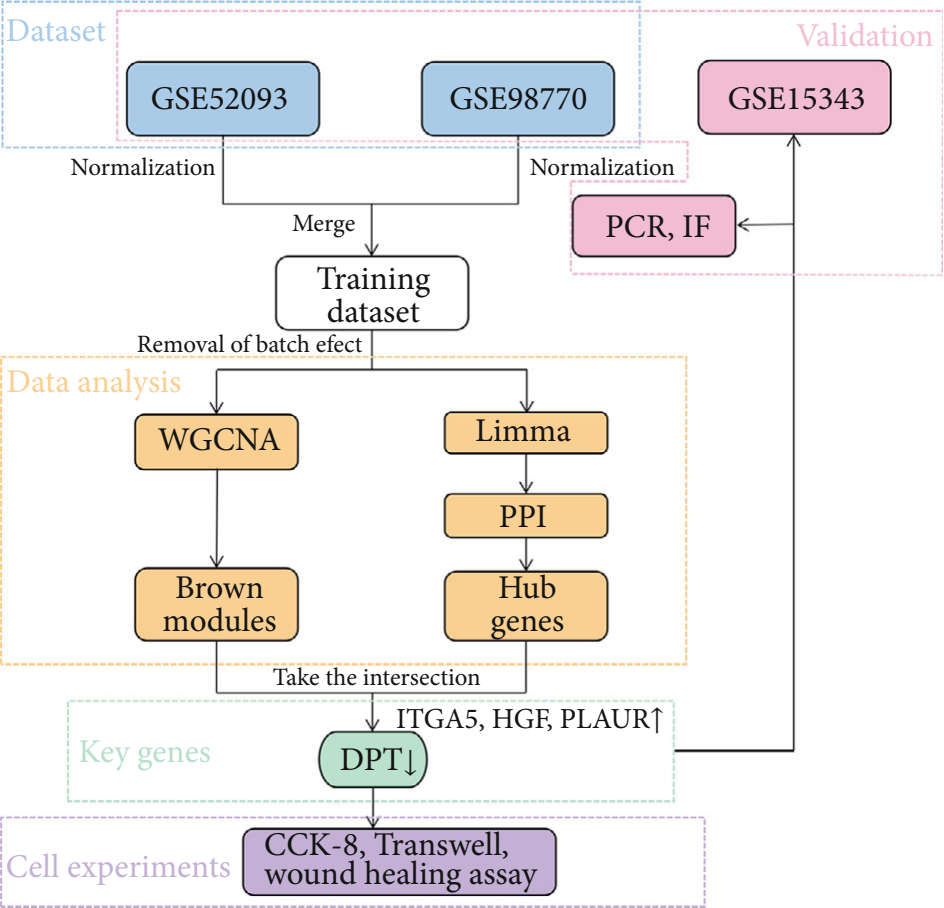
Workflow of the study design.

Key ATAAD‐related genes were identified by overlapping key modules and hub genes. The protein encoded by the DPT gene is a component of the extracellular matrix (ECM) and regulates interactions among ECM proteins. It is also associated with a variety of cancers, where it appears to be involved in tumor metastasis, as well as ventricular remodeling and other diseases. Cell Counting Kit 8 (CCK‐8), Transwell, and wound healing assays were used to examine the effect of DPT on the function of HASMC. The study workflow is illustrated in Figure 1. This study ultimately identified four key genes associated with ATAAD, confirming that DPT regulates HASMC function and provides a basis for further efforts to establish diagnostic biomarkers and therapeutic targets in ATAAD.

## 2. Materials and Methods

### 2.1. Dataset Selection and Preprocessing

Three publicly available microarray datasets—GSE52093, GSE98770, and GSE153434—were retrieved from the GEO database to explore gene expression differences for ATAAD. These datasets included human aortic tissue samples, standard controls, and ATAAD patients. GSE52093 consisted of 5 control and 7 ATAAD samples, GSE98770 corresponded to 5 control and 6 ATAAD samples (only mRNA data were used for this), and GSE153434 provided 10 control and 10 ATAAD samples for independent validation. Probe IDs were converted to gene symbols using the platform‐specific probe annotation files. The gene with the highest expression value was selected from the probes representing it. The merged training dataset (GSE52093 and GSE98770) was cleaned for batch effects using the svart package in R. The data were then merged and normalized to uniform expression levels across samples.

### 2.2. Differential Gene Expression Analysis

DEGs were identified between ATAAD samples and normal using the “limma” package in R. Genes were filtered with an adjusted *p* value (i.e., false discovery rate [FDR]) less than 0.05 and an absolute log2 fold change (|log2FC|) greater than or equal to 1. This led to the identification of both up‐ and downregulated genes. Downstream functional enrichment and network analyses were performed using these DEGs.

### 2.3. WGCNA

WGCNA was applied to the training dataset to identify essential gene modules associated with ATAAD [9]. First, a sample clustering analysis showed no significant outliers. To obtain a scale‐free network topology (*R* ^2^ > 0.9), a soft‐thresholding power (*β* = 16) was selected. Hierarchical clustering and dynamic tree cutting were used with a minimum module size of 40 to group genes into modules. The module had been given a unique color identifier. We identified the module most strongly correlated with the disease by calculating the correlation between the module eigengenes and the ATAAD trait. For further analysis, the module with the highest correlation was selected.

### 2.4. GO and KEGG Pathway Enrichment Analyses

To gain functional insight into the identified DEGs, the DEGs were subjected to GO enrichment analysis and KEGG enrichment analysis using the “clusterProfiler” package in R [10, 11]; this enriched the DEGs in biological processes (BPs), cellular components (CCs), and molecular functions (MFs). Relevant signaling pathways for ATAAD pathogenesis could be identified by KEGG analysis. GO terms and pathways with adjusted *p* values < 0.05 were significantly enriched. GraphPad Prism 9 was used to visualize the results.

### 2.5. PPI Network and Hub Gene Identification

A PPI network was constructed using the STRING database to identify central genes within the DEG network [12]. For the interaction confidence score, we chose 0.4 as the threshold. The resulting network was visualized and analyzed via Cytoscape [13]. The Top 30 hub genes, ranked by node connectivity, were identified using the CytoHubba plugin [14]. We then intersected these hub genes with the critical WGCNA module to identify the overlapping genes most pertinent to ATAAD.

### 2.6. Collection and Processing of Human Aortic Tissue Samples

Human aortic tissue was collected from patients undergoing open aortic replacement for ATAAD and from control patients undergoing coronary artery bypass grafting (CABG) without aortic lesions ruled out by imaging and surgical evaluation. Within 30 min after resection, tissues were harvested, rinsed in cold saline to remove blood and thrombi, and snap‐frozen in liquid nitrogen; they were stored at −80°C for use. All samples were obtained in accordance with the proper ethical protocol.

### 2.7. Collection and Processing of Human Tissue Samples

All patients provided written informed consent before sample collection. Inclusion criteria for the ATAAD group included (1) diagnosis confirmed by CT angiography or intraoperative findings, (2) no prior aortic surgery, and (3) absence of active infection, autoimmune disease, or malignancy. Control samples were obtained from patients undergoing CABG with standard aortic structure confirmed by preoperative imaging and intraoperative assessment. Collection and handling of tissue and green samples were performed under sterile conditions within 30 min of excision, in accordance with approved institutional protocols. This study was approved by the Ethics Committee of the First Affiliated Hospital of Bengbu Medical University (Approval No. 2018039), and all procedures adhered to the Declaration of Helsinki.

### 2.8. Quantitative Real‐Time PCR (qPCR) Analysis

ATAAD and control aortic tissues 14 cases were dissected fresh, and total RNA was extracted using TRIzol reagent, followed by chloroform extraction, isopropanol precipitation, and ethanol washes. The dissolved RNA in DEPC‐treated water was stored at −80°C. The concentration of RNA was measured with a spectrophotometer, and cDNA synthesis was performed with NovoScript Plus All‐in‐One 1st Strand cDNA Synthesis SuperMix (Novoprotein, Shanghai). The Novo Start SYBR qPCR SuperMix Plus kit was used for qPCR, with GAPDH as the reference gene. The relative expression levels of target genes were calculated by the 2^−*Δ* *Δ*Ct^ method. Table 1 lists the primer sequences used.

**Table 1:** Details for the acute Type A aortic dissection microarray study.

**Dataset**	**Sample**	**Platform**	**Normal**	**ATAAD**	**Contributor**
GSE52093	Ascending aorta tissue	GPL10558	5	7	Pan
GSE98770	Ascending aorta tissue	GPL14550	5	6	Futamura et al.
GSE153434	Ascending aorta tissue	GPL20795	10	10	Zhou et al.

### 2.9. Immunofluorescence Analysis

Formalin‐fixed, paraffin‐embedded aortic tissue sections from five ATAAD and five control patients were immunofluorescence‐stained. After deparaffinization, antigen retrieval was performed, and the nonspecific binding was blocked with BSA. Primary antibodies against DPT, ITGA5, hepatocyte growth factor (HGF), and PLAUR (plasminogen activator urokinase receptor) (all Affinity, 1:100) were added to sections and incubated overnight at 4°C. Sections the next day were incubated with cyanine3 (Cy3)‐conjugated secondary antibodies (Servicebio, 1:300) for 50 min at 37°C. Nuclear staining was accomplished with DAPI. This autofluorescence was quenched before mounting, and the sections were imaged with a Nikon confocal laser‐scanning microscope. In three randomly selected fields per sample, fluorescence intensity was quantified using ImageJ.

### 2.10. Cell Culture and siRNA Transfection

Primary HASMCs were obtained from Priscilla (Wuhan, China). They were cultured in smooth muscle cell medium supplemented with 5% fetal bovine serum, 1% penicillin–streptomycin, growth supplement, and insulin. In a humidified 5% CO₂ incubator, cells were maintained at 37°C, and cells between Passages 3 and 6 were included in experiments. Cells were then transfected with either control siRNA (NC group) or DPT‐specific siRNA (si‐DPT group) using HANBIO reagents (Shanghai, China) according to the manufacturer’s protocol. Cells used in all experiments were passage‐matched (Passages 3–4) to minimize passage‐related phenotypic drift.

### 2.11. Western Blot

Total protein was extracted using RIPA lysis buffer supplemented with protease inhibitors. Protein was separated by SDS‐PAGE and transferred onto PVDF membranes. After blocking with 5% BSA for 1 h at room temperature, membranes were incubated overnight at 4°C with primary antibodies against DPT (1:1000, [Abcam, Cat: ab255823]) and *β*‐actin (1:1000, [CST, Cat: #4967]) as a loading control. HRP‐conjugated secondary antibodies (1:3000, [CST, Cat#7074]) were applied for 1 h at room temperature, and protein bands were visualized using an enhanced chemiluminescence (ECL) system. Densitometric analysis was performed using ImageJ software, and DPT expression was normalized to *β*‐actin.

### 2.12. Wound Healing Assay

Cell migration of the control (NC) and DPT knockdown (si‐DPT) groups was assessed using a scratch wound healing assay. Left: Representative phase‐contrast images of scratch wounds at 0, 24, and 48 h (red lines indicate wound edges; scale bar, 200 *μ*m). Right: Quantitative analysis of wound healing rate. Data are presented as mean ± SD (**n** = 3 per group per time point), with normal distribution confirmed by the Shapiro‐Wilk test (*p* > 0.05) and homogeneous variance confirmed by Levene’s test (*p* > 0.05). Statistical analysis was performed using two‐way ANOVA (main effect of group: *F* = 42.67, *p* < 0.0001; main effect of time: *F* = 89.32, *p* < 0.0001; interaction effect: *F* = 15.84, *p* < 0.001) followed by post hoc two‐sample comparisons (Bonferroni‐corrected unpaired Student’s **t**‐test).  ^∗∗∗∗^ Adjusted *p* < 0.0001 versus NC group at the same time point.

### 2.13. Cell Proliferation Assay (CCK‐8)

The CCK‐8 (GlpBio, Shanghai) was used to assess cell proliferation. Five times 10^4^ cells per milliliter in the logarithmic growth phase were seeded into 96‐well plates. At 12, 24, and 48 h, 100 *μ*L CCK‐8 solution was added to each well and incubated for 1 h. Proliferation rates were determined by measuring absorbance at 450 nm with a microplate reader. All experiments were performed in triplicate and independently repeated at least three times.

### 2.14. Transwell Migration Assay

For the migration assay, 100 *μ*L of cell suspension (2 × 10^5^ cells/mL) was seeded into the upper chamber of 24‐well Transwell inserts, and 600 *μ*L of complete medium was added to the lower chamber to measure cell migration. After 24 h of incubation, the upper side cells were removed, and the underside cells that migrated were fixed with paraformaldehyde and stained with 0.1% crystal violet. Migrated cell counts were calculated using ImageJ, and images were captured under a microscope. All experiments were performed in triplicate and independently repeated at least three times.

### 2.15. Statistical Analysis

All statistical analyses were performed using GraphPad Prism 9. First, the Shapiro‐Wilk test was applied to assess the normality of continuous variables: For constant data that conformed to a normal distribution (Shapiro‐Wilk test, *p* > 0.05), results were expressed as mean ± SD (SD), and intergroup comparisons were conducted using an unpaired Student’s **t**‐test (for equal variance, verified by the **F**‐test) or Welch’s **t**‐test (for unequal variance); for continuous data that did not conform to a normal distribution (Shapiro‐Wilk test, *p* ≤ 0.05), results were strictly expressed as median (interquartile range [IQR]) (instead of mean ± SD), and intergroup comparisons were performed using the nonparametric Mann–Whitney **U** test. The diagnostic predictive value of candidate genes was evaluated using receiver operating characteristic (ROC) curve analysis, with statistical significance defined as *p* < 0.05.

## 3. Results

### 3.1. Study Workflow Overview

Figure 1 illustrates the complete analytical pipeline, outlining the study’s overall design. The workflow starts with retrieving and normalizing two microarray datasets (GSE52093 and GSE98770). It proceeds to differential gene expression analysis, functional enrichment analysis, the construction of a PPI network, WGCNA, validation on an independent dataset (GSE153434), and experimental verification using qPCR, immunofluorescence, and functional cellular assays.

### 3.2. Dataset Normalization and Batch Effect Removal

We normalized the raw expression profiles of GSE52093 and GSE98770, removed batch effects, and combined them into a unified training dataset to guarantee comparability across datasets. Figures 2a, 2b, 2c, and 2d show the normalization of GSE52093 and GSE98770 separately, and Figure 2e,f shows batch effect correction of normalized data from GSE52093 and GSE98770 combined. These preprocessing steps were crucial to reducing technical variance and amplifying the biological signal of interest.

Figure 2Normalization of GSE52093 and GSE98770 and batch removal of the training dataset. (a, b) Normalization of dataset GSE52093. (c, d) Normalization of dataset GSE98770. (e, f) Batch removal of the training dataset.

**Figure:**
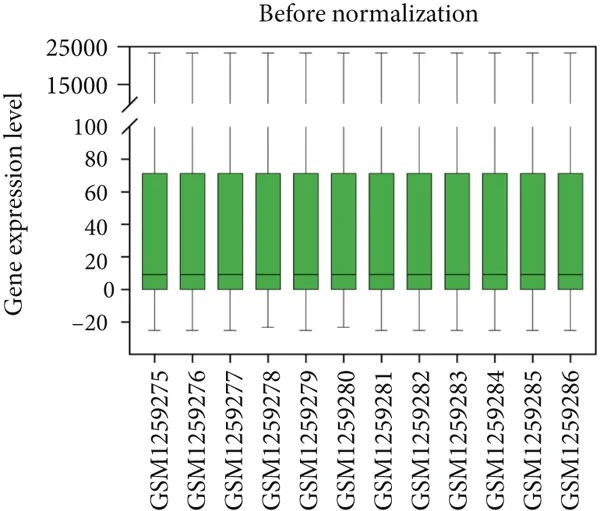
(a)

**Figure:**
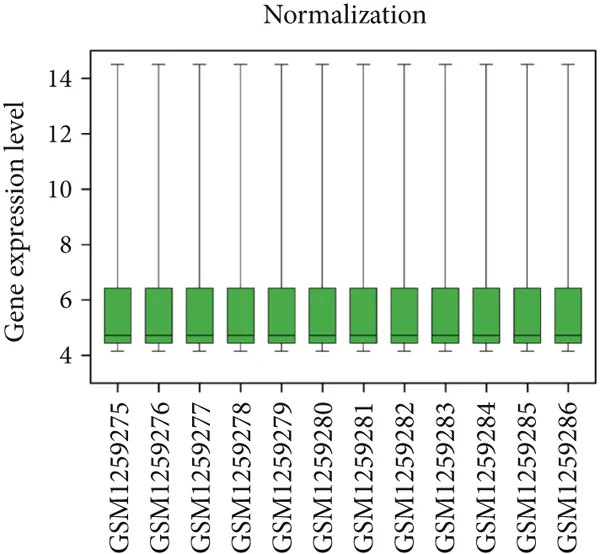
(b)

**Figure:**
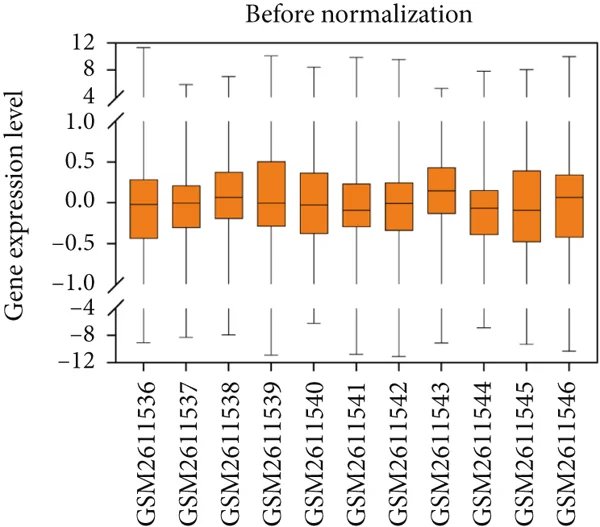
(c)

**Figure:**
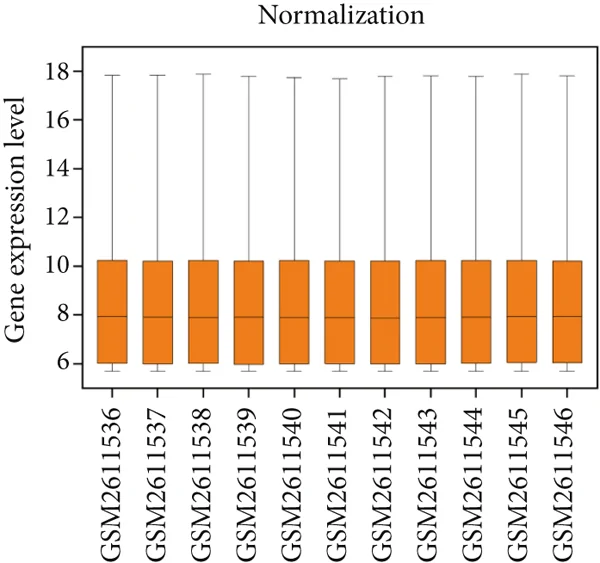
(d)

**Figure:**
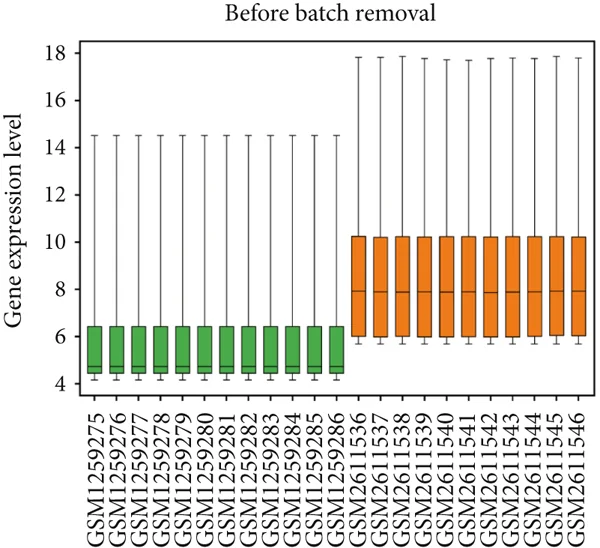
(e)

**Figure:**
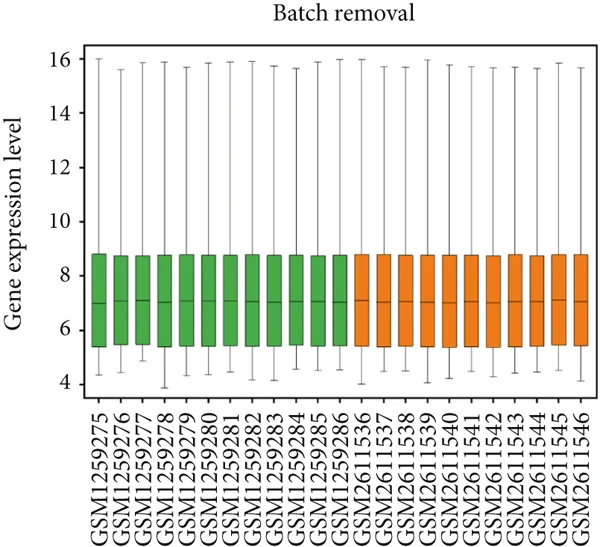
(f)

### 3.3. Identification of DEGs

Normalization of raw data revealed 441 DEGs as defined by adjusted *p* value < 0.05 and |log2 fold − change| ≥ 1.0, using the limma R package (Table S1). Of these, 164 genes were significantly upregulated in ATAAD samples compared to controls, and 277 were significantly downregulated. The volcano plot (Figure 3a) shows the distribution of these DEGs, with significant genes indicated in red (upregulated) or green (downregulated). Moreover, the ATAAD versus control comparisons of the Top 60 DEGs are shown in a heat map (Figure 3b), revealing distinct expression patterns between the ATAAD and control groups.

Figure 3Volcano plot and heat map of all differentially expressed genes (DEGs). (a) Volcano plot: DEGs were identified using Student’s *t*‐test (adjusted *p* < 0.05, |log_2_FC| ≥ 1.0) via the “limma” package in R. This test was selected because the normalized data of the merged training datasets (GSE52093 + GSE98770) passed the normality test. (b) Heat map of the Top 60 DEGs: Expression values were normalized and conformed to a normal distribution; the visualization of intergroup difference trends is based on the results of Student’s *t*‐test.

**Figure:**
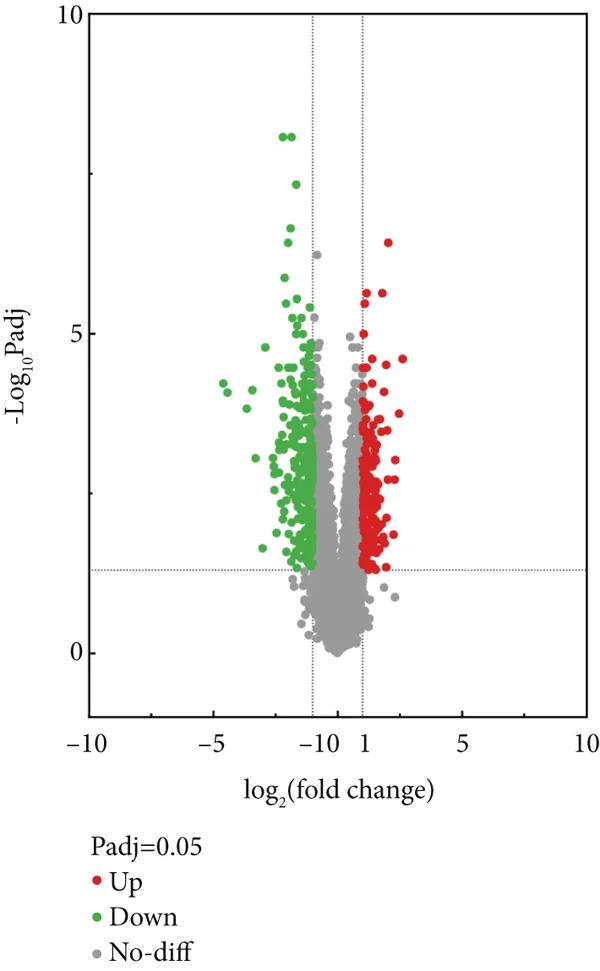
(a)

**Figure:**
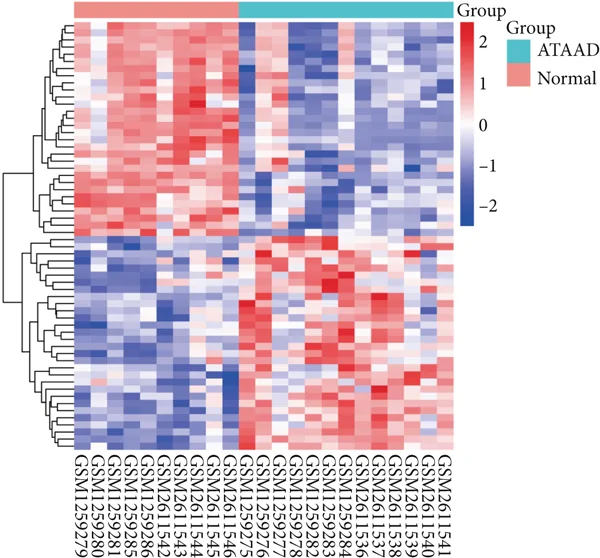
(b)

### 3.4. Construction of Coexpression Network and Module Identification

Based on WGCNA, it was applied to the training dataset to identify coexpressed gene modules. The sample was also confirmed to be free of outliers by sample clustering (Figure 4a). To satisfy the scale‐free topology criterion (*R* ^2^ > 0.9) (Figure 4b), a soft‐thresholding power of *β* = 16 was chosen. Using this threshold, genes were clustered into 26 modules via dynamic tree cutting (Figure 4c).

Figure 4Construction of a weighted gene coexpression network analysis network. (a) Cluster analysis and trait heat map for each sample of the training dataset. (b) Suitable soft threshold powers selection, the red line indicating 0.9. (c) The cluster dendrogram of genes in the weighted gene coexpression network analysis.

**Figure:**
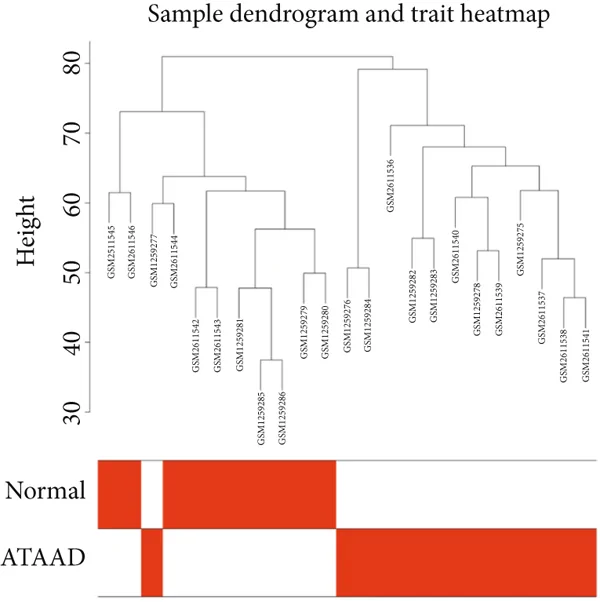
(a)

**Figure:**
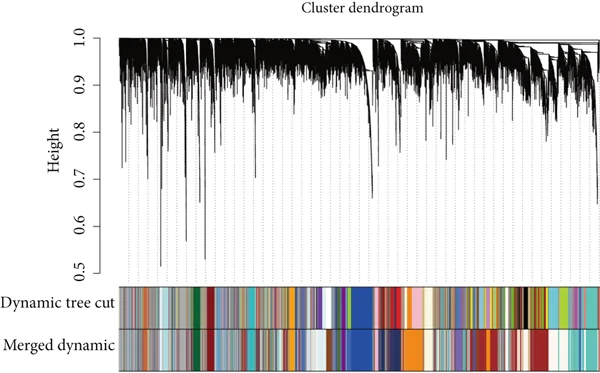
(b)

**Figure:**
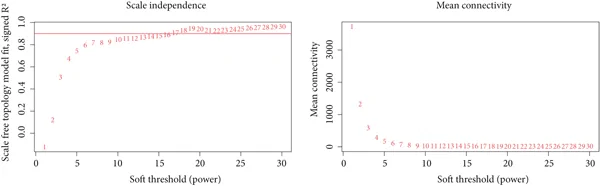
(c)

An adjacency heat map of relationships between module eigengenes (Figure 5a) and a heat map of correlations between modules and the ATAAD trait (Figure 5b) were employed. The brown module was the most strongly correlated with ATAAD (correlation coefficient = 0.68, *p* < 0.001). Finally, in a scatterplot of gene significance versus module membership (Figure 5c), we found that highly connected module genes were also highly correlated with ATAAD.

Figure 5Identification of the key module. (a) Eigengene adjacency heat map of different modules. (b) Heat map of the correlation between status (ATAAD and normal) and module eigengenes. (c) Scatterplot of connectivity versus GS for ATAAD of modules.

**Figure:**
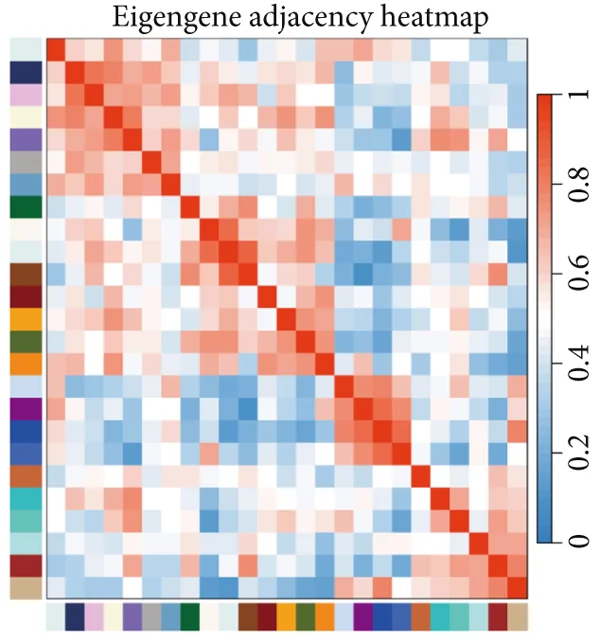
(a)

**Figure:**
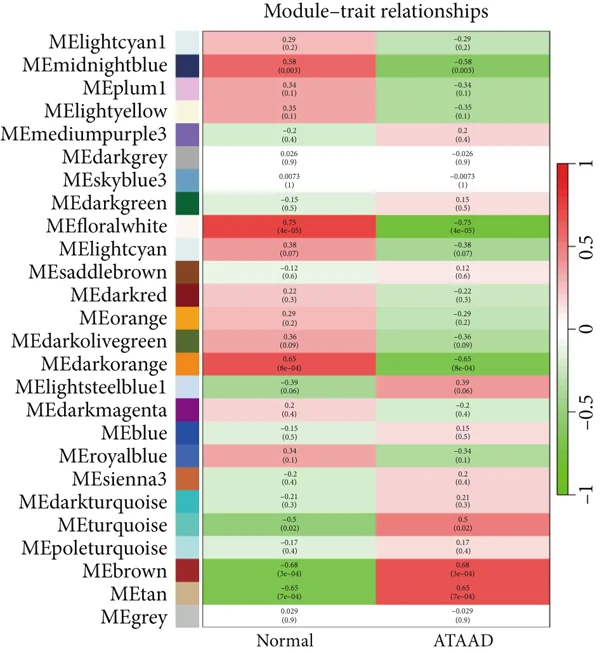
(b)

**Figure:**
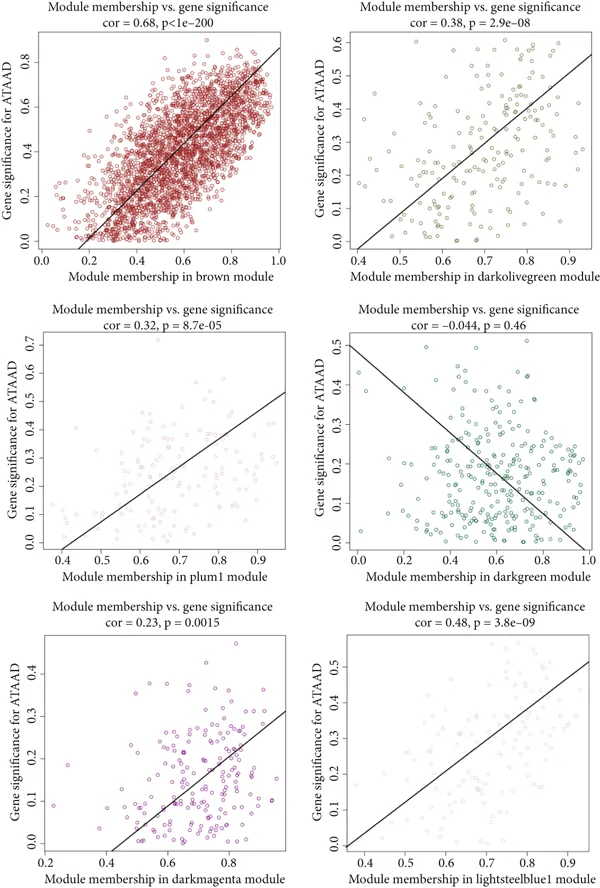
(c)

### 3.5. GO and KEGG Pathway Enrichment Analyses

GO and KEGG enrichment analyses were performed to explore the biological significance of the DEGs. DEGs were significantly enriched in BPs such as muscle system processes, actin filament organization, and regulation of cell adhesion, as shown in Figures 6a, 6b, and 6c. The MFs were actin binding, integrin binding, and ECM structural constituents. Enrichment was observed for CC terms in focal adhesions, sarcomeres, and the collagen‐containing ECM.

Figure 6Gene Ontology and Kyoto Encyclopedia of Genes and Genomes analyses of DEGs. (a–c) Gene Ontology analyses of DEGs. (d) Kyoto Encyclopedia of Genes and Genomes analyses of DGEs.

**Figure:**
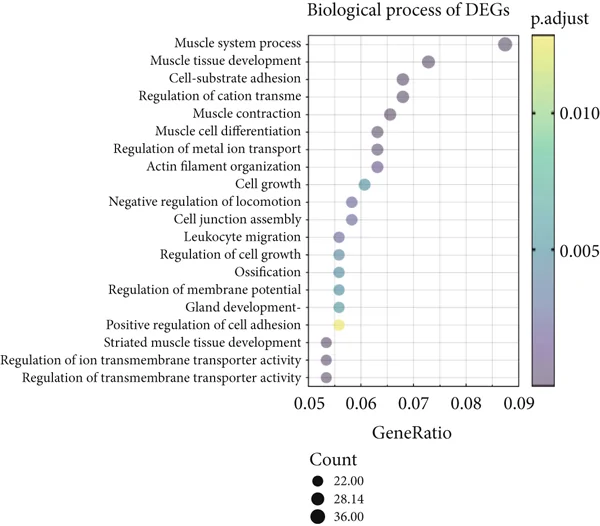
(a)

**Figure:**
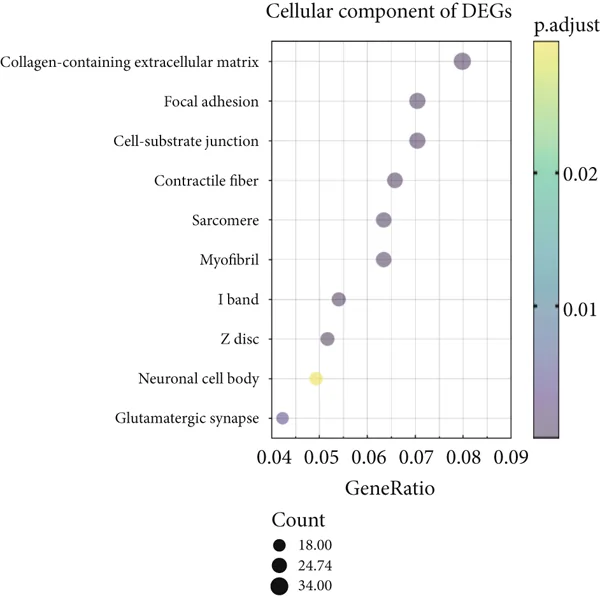
(b)

**Figure:**
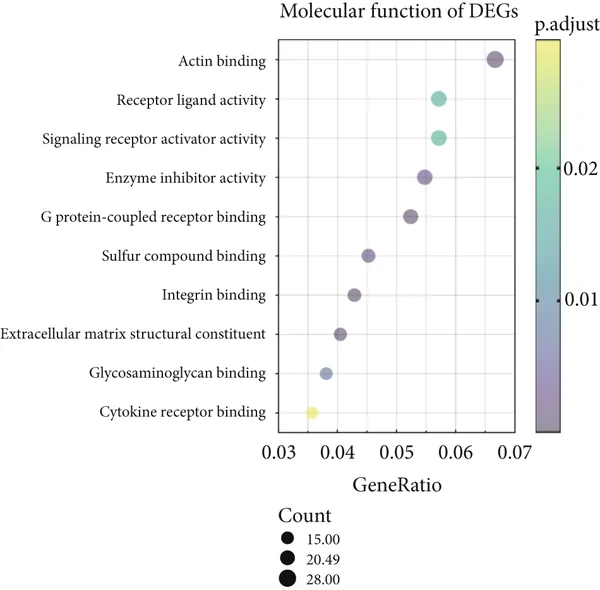
(c)

**Figure:**
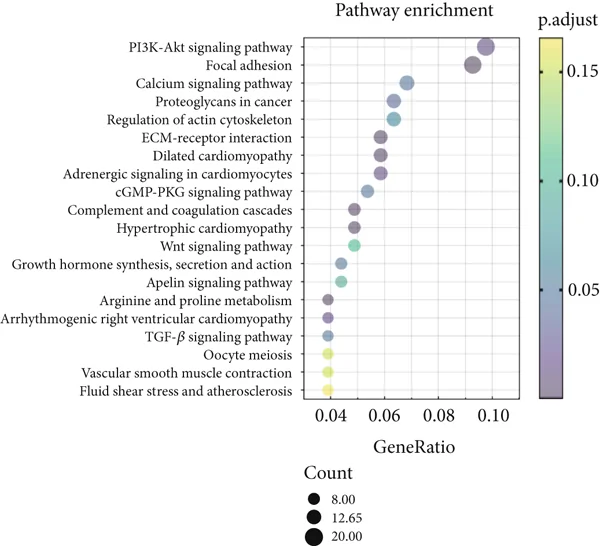
(d)

KEGG pathway analysis (Figure 6d) showed significant enrichment in signaling pathways involved in aortic pathology, including PI3K‐Akt signaling, ECM‐receptor interaction, focal adhesion, cGMP‐PKG signaling, and TGF‐beta signaling. These pathways involve vascular smooth muscle cell (VSMC) function, matrix remodeling, and aortic wall integrity.

### 3.6. Hub Gene Identification via PPI Network and Module Overlap

The 441 DEGs were used to construct a PPI network using STRING. The resulting network is shown in Figure 7a and Table S2, with each node representing a gene and the node size and color intensity corresponding to the interaction degree. The most connected node was assigned to VEGFA. CytoHubba was then used to identify the Top 30 hub genes (Figure 7b), excluding GAP43, which was deemed irrelevant. A Venn diagram (Figure 7c) was used to visualize the intersection of these top hub genes with the genes in the brown WGCNA module and to show that four overlapping genes—DPT, ITGA5, HGF, and PLAUR—exist, which are key candidates for further validation.

Figure 7The intersection of the brown module and the Top 30 hub genes was found to identify key genes. (a) PPI network analyses of DEGs: Each circle represents a gene, and the darker the color and the larger the circle, the greater the number of associations. Most significant hub genes, according to the highest number of connections, are arranged in the innermost circle. (b) The Top 30 hub genes were detected from the PPI network by CytoHubba (removing a not related gene GAP43). (c) Venn diagram.

**Figure:**
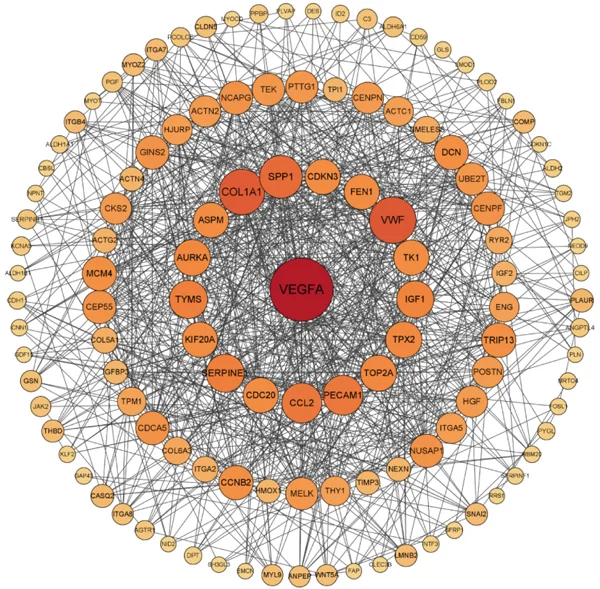
(a)

**Figure:**
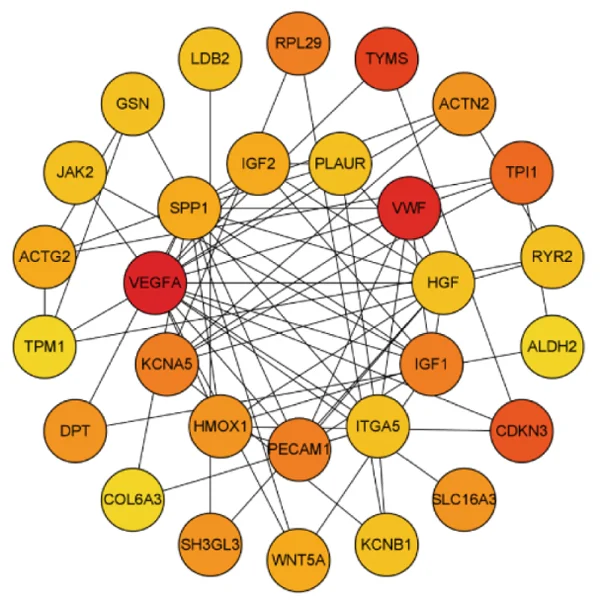
(b)

**Figure:**
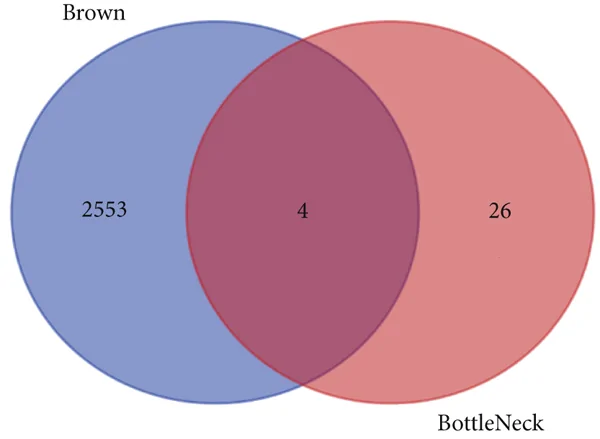
(c)

### 3.7. Validation of Key Gene Expression

The four key genes were validated in a training set and the GSE153434 dataset, where expression of ITGA5, HGF, and HGF was found to be upregulated and DPT was downregulated in ATTAD compared to normal in both datasets. Figure 8a shows that DPT was significantly downregulated, and ITGA5, HGF, and PLAUR were upregulated considerably in the training dataset (ATAAD). The qPCR analysis of 14 ATAAD and control aortic tissue specimens (Figure 8b) confirmed these patterns. The results of the ROC curve analysis in the GSE153434 dataset (Figure 8c) showed high predictive accuracy (AUC = 0.99 for ITGA5, 0.83 for HGF, 0.96 for PLAUR, and 0.96 for DPT) and strong diagnostic potential.

Figure 8Validation of key gene expression. (a) Expression of key genes in the training datasets (GSE52093 + GSE98770): The data conformed to a normal distribution, so intergroup comparisons were performed using Student’s *t*‐test (DPT: *t* = 6.23, *p* < 0.0001; ITGA5: *t* = 5.81, *p* < 0.0001; HGF: *t* = 4.92, *p* < 0.001; PLAUR: *t* = 5.37, *p* < 0.0001). (b) Expression of four key genes in normal (*n* = 14) and ATAAD (*n* = 14) aortic tissue (qPCR): The data conformed to a normal distribution, so intergroup comparisons were performed using Student’s *t*‐test (DPT: *t* = 7.15, *p* < 0.0001; ITGA5: *t* = 6.03, *p* < 0.0001; HGF: *t* = 5.28, *p* < 0.001; PLAUR: *t* = 5.72, *p* < 0.0001). (c) ROC curve analyses of key genes in the GSE153434 dataset: AUC values were calculated based on gene expression data (which conformed to a normal distribution; intergroup differences were first verified by Student’s *t*‐test, *p* < 0.001) to evaluate diagnostic value.  ^∗^ *p* < 0.05;  ^∗∗∗^ *p* < 0.001;  ^∗∗∗∗^ *p* < 0.0001.

**Figure:**
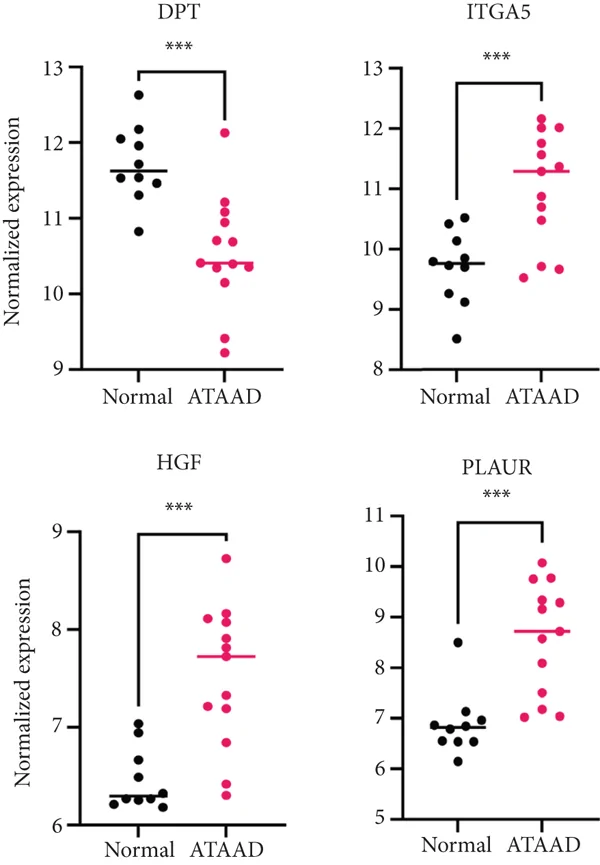
(a)

**Figure:**
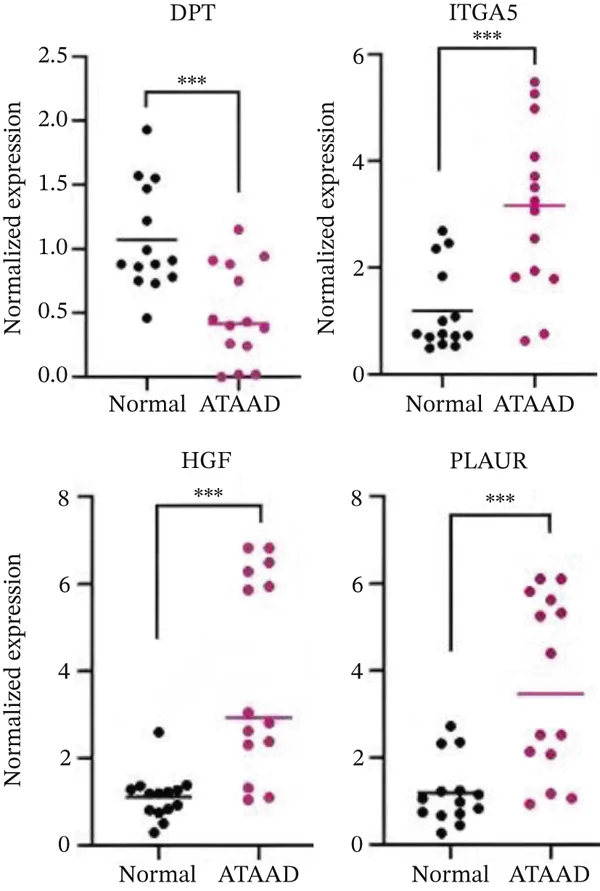
(b)

**Figure:**
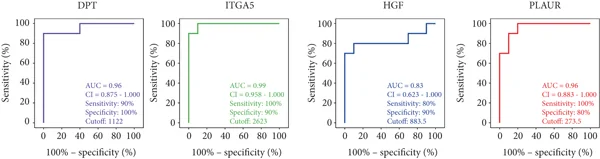
(c)

### 3.8. Immunofluorescence‐Based Validation

Immunofluorescence staining was used for protein‐level validation. Representative staining images for all four genes (Figure 9) are shown in normal and ATAAD tissues. The relative mean fluorescence intensity of DPT was significantly lower in ATAAD tissues than in normal tissues (mean ± SD: 0.32 ± 0.11 vs. 1.00 ± 0.15, *p* < 0.01), while ITGA5 (1.85 ± 0.22 vs. 1.00 ± 0.18, *p* < 0.01), HGF (1.68 ± 0.19 vs. 1.00 ± 0.16, *p* < 0.05), and PLAUR (1.76 ± 0.21 vs. 1.00 ± 0.17, *p* < 0.01) were significantly higher. Protein expression is green fluorescence; nuclei were counterstained with DAPI (blue). These data were confirmed by quantitative analysis of mean fluorescence intensity, with statistically significant differences between groups.

Figure 9Immunofluorescent validation of key proteins in normal (*n* = 5) and ATAAD (*n* = 5) tissues. (a) Representative staining images (DPT: red fluorescence; nuclei: DAPI, blue). (b) Quantitative analysis of mean fluorescence intensity. The data of DPT, ITGA5, HGF, and PLAUR did not conform to a normal distribution, so intergroup comparisons were performed using the Mann–Whitney U test (DPT: *U* = 2.0, *p* < 0.01; ITGA5: *U* = 3.0, *p* < 0.01; HGF: *U* = 4.0, *p* < 0.05; PLAUR: *U* = 2.5, *p* < 0.01).  ^∗^ *p* < 0.05;  ^∗∗^ *p* < 0.01;  ^∗∗∗^ *p* < 0.001. Scale bar, 100 *μ*m.

**Figure:**
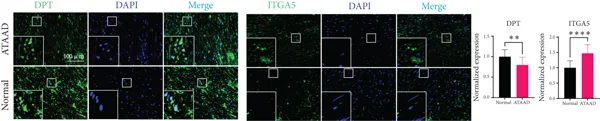
(a)

**Figure:**
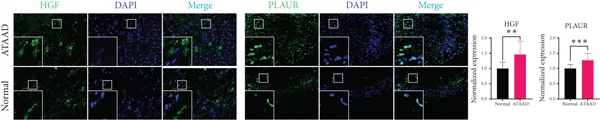
(b)

### 3.9. Functional Validation Through Cell‐Based Assays

In vitro knockdown experiments were performed using siRNA to investigate the functional role of DPT in aortic smooth muscle cells. Additionally, we have now included Western blot validation data to confirm siRNA knockdown efficiency at the protein level (Figure S1). Wound healing assay to detect cell migration of the NC and si‐DPT groups. Adjusted *p* < 0.0001 versus NC group at the same time point (Figure 10a) caused significantly greater cell migration than controls. Consistent with this, the number of migrated HASMCs in the si‐DPT group was considerably higher than that in the NC group (median [IQR]: 125 [118‐135] vs. 58 [50‐65], U = 0.0, *p* < 0.0001) (Figure 10b). si‐DPT cells showed enhanced transmembrane movement. In contrast, DPT knockdown decreased cellular proliferation, as demonstrated by the CCK‐8 assay at 12, 24, and 48 h (Figure 10c). These results indicate that DPT plays a critical role in regulating HASMC proliferation and migration, which are essential to aortic wall physiology.

Figure 10Functional validation of DPT in HASMCs. (a) Wound healing assay (cell migration): NC group (*n* = 3) and si‐DPT group (*n* = 3). The wound healing rate data conformed to a normal distribution, allowing for intergroup comparisons using Student’s *t*‐test (24 h: *t* = 5.68, *p* < 0.001; 48 h: *t* = 7.21, *p* < 0.0001): scale bar, 200 *μ*m. (b) Transwell assay (cell migration): The number of migrated cells did not conform to a normal distribution, so intergroup comparisons were performed using the Mann–Whitney *U* test (*U* = 0.0, *p* < 0.01): scale bar, 100 *μ*m. (c) CCK‐8 assay (cell proliferation): The absorbance values (450 nm) conformed to a normal distribution, so intergroup comparisons were performed using Student’s *t*‐test (12 h: *t* = 3.85, *p* < 0.01; 24 h: *t* = 6.02, *p* < 0.001; 48 h: *t* = 8.15, *p* < 0.0001).  ^∗∗^ *p* < 0.01;  ^∗∗∗^ *p* < 0.001;  ^∗∗∗∗^ *p* < 0.0001.

**Figure:**
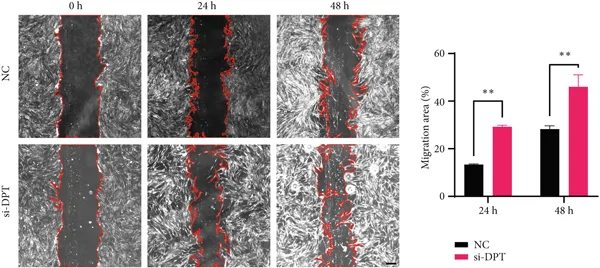
(a)

**Figure:**
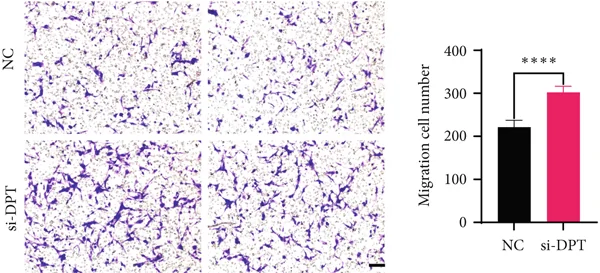
(b)

**Figure:**
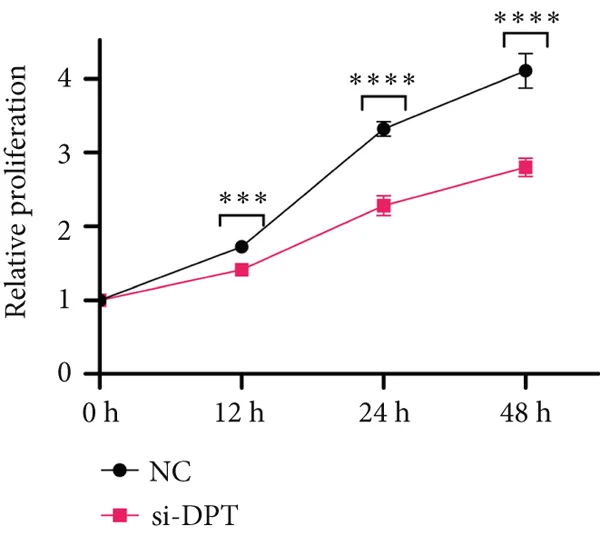
(c)

### 3.10. Dataset and Primer Information

To provide transparency and reproducibility, Table 2 lists the three datasets used in this study, their platforms, sample sizes, and origin contributors. Sequences of forward and reverse primers used for qPCR analysis of DPT, ITGA5, HGF, PLAUR, and the housekeeping gene GAPDH are shown in Table 1. These technical details enable future studies to reproduce the validation assays.

**Table 2:** Sequences of forward and reverse primers used for quantitative real‐time PCR (qPCR) validation of key genes (DPT, ITGA5, HGF, PLAUR) and the housekeeping gene GAPDH.

**Gene**	**Forward**	**Reverse**
**GAPDH**	5 ^′^‐CAGGAGCATTGCTGATGAT‐3 ^′^	5 ^′^‐GAAGGCTGGGGTCATTT‐3 ^′^
VEGFA	5 ^′^‐AGGGCAGAATCATCACGAAGT‐3 ^′^	5 ^′^‐AGGGTCTCGATTGGATGGCA‐3 ^′^
ITGA5	5 ^′^‐GGCTTCAACTTAGACGCGGAG‐3 ^′^	5 ^′^‐TGGCTGGTATTAGCCTTGGGT‐3 ^′^
HGF	5 ^′^‐GCTATCGGGGTAAAGACCTACA‐3 ^′^	5 ^′^‐CGTAGCGTACCTCTGGATTGC‐3 ^′^
PLAUR	5 ^′^‐TGTAAGACCAACGGGGATTGC‐3 ^′^	5 ^′^‐AGCCAGTCCGATAGCTCAGG‐3 ^′^
DPT	5 ^′^‐GGGGCCAGTATGGCGATTATG‐3 ^′^	5 ^′^‐CGGTTCAAATTCACCCACCC‐3 ^′^

## 4. Discussion

Although advances in surgical technique and perioperative management have been made, ATAAD is still a devastating cardiovascular emergency characterized by abrupt onset, rapid progression, and high mortality. Currently, diagnostic practices remain heavily dependent on imaging modalities, which, although effective, are limited in availability, costly, and require transferring patients to a tertiary care facility (especially in emergencies). Furthermore, due to the lack of reliable molecular biomarkers and effective pharmacological therapies targeting ATAAD, early detection and personalized treatment are currently limited. To fill this gap, this study is aimed at integrating high‐throughput gene expression data with in vitro validation to identify novel molecular diagnostic and therapeutic targets for ATAAD, with a focus on DPT.

Degeneration of the aortic medial layer is the histopathological hallmark of ATAAD, which is characterized by fragmentation of elastic fibers, loss of VSMCs, and disorganization of ECM components [15, 16]. These changes compromise the aorta’s structural and mechanical integrity, rendering it susceptible to dissection under physiological pressure. VSMC s, including HASMCs, are essential for maintaining ECM homeostasis through the (de)synthesis and remodeling of collagen, elastin, and proteoglycans. In aortic diseases [17], their dysfunction is a major contributor to medial degeneration. However, regarding ECM remodeling in aortic pathology, our identification of DPT as a downregulated gene in ATAAD and our further characterization of its role in regulating HASMC migration and proliferation provide a novel layer of understanding in this system.

Dermatopontin (DPT) is a tyrosine‐rich noncollagenous ECM protein that has been described to interact with decorin and other matrix components to regulate collagen fibrillogenesis and matrix assembly [18, 19]. Cutaneous elasticity and wound healing have previously been implicated; cardiovascular remodeling and oncogenesis are being seen as roles for their increase. Furthermore, DPT influences TGF‐*β* activation [20, 21]. However, it modulates integrin signaling and augments cellular adhesion to the ECM. Its tumor‐suppressive activity has been studied in hepatocellular carcinoma giant cell tumors bone and other tumors of which are often downregulated in metastatic lesions [22–26]. Interestingly, this was found to stimulate cell migration and inhibit cell adhesion in our study by downregulating DPT in DPT knockdown specimens, an effect also observed in wound healing and Transwell assays in HASMCs.

As a dynamic reservoir of biochemical and biomechanical signals, the ECM modulates VSMC behavior. Inflammatory disruption of ECM integrity can form a vicious cycle of inflammation, proteolysis, and apoptosis, further weakening the aortic wall [17]. In ATAAD tissue, reduced DPT expression is observed. DPT‐deficient HASMCs exhibit decreased proliferation and increased migration, suggesting that DPT is a structural and regulatory molecule that protects against pathological remodeling. This agrees with previous results showing that the dermatopontin can leads to changes in the elasticity of the mice skin and the accumulation of collagen [19]. Moreover, DPT was upregulated in response to hypoxic stress in neonatal rat cardiomyocytes, implicating DPT in ischemia‐related ventricular remodeling [27].

Further, three other significant upregulated genes (i.e., ITGA5, HGF, and PLAUR) were identified in our study. ITGA5 is a member of the integrin *α* subfamily, which is critical for cell–ECM interactions and influences cell adhesion, migration, and possibly cell survival. Various cancers have been shown to activate PI3K/Akt and ERK/MAPK downstream signaling pathways, thereby inducing angiogenesis and tissue remodeling [28, 29]. The phenotypic effects observed following DPT knockdown—namely, enhanced HASMC migration and reduced proliferation—are consistent with the BPs and signaling pathways identified in our enrichment analyses. Pathways such as PI3K‐Akt, focal adhesion, ECM‐receptor interaction, and TGF‐*β* signaling play central roles in regulating smooth muscle cell behavior, cytoskeletal dynamics, and ECM remodeling. These results suggest that DPT may modulate VSMC function, at least in part, by influencing these key molecular cascades. Moreover, ITGA5 overexpression in vascular tissue correlates with aneurysm formation and inflammatory infiltration; it is hypothesized that ITGA5 plays a pathogenic role in vascular wall instability [30, 31]. We demonstrated in prior studies that ITGA5 expression is high in ATAAD tissue and correlates with disease status.

A key player in vascular biology is the HGF, which is widely known for its regenerative and mitogenic effects on the c‐Met receptor. It increases endothelial cell proliferation, induces angiogenesis, and controls inflammation [32, 33]. Expression of HGF has been observed to be high in cardiovascular diseases such as atherosclerosis and myocardial infarction [34, 35]. In ATAAD, elevated HGF levels may be a compensatory response aimed at promoting vascular repair. However, the ultimate balance of regeneration versus pathological remodeling has not been defined.

Our study also identifies PLAUR (uPAR) as another upregulated gene. ECM degradation by activating the plasminogen–plasmin system plays a central role in tissue remodeling, cell migration, invasion, and other processes and is the main activity of PLAUR [36]. In AD and aneurysmal disease, high PLAUR expression is associated with increased proteolytic activity and inflammatory cell infiltration [36, 37]. Further experimental evidence for PLAUR’s involvement in ATAAD pathogenesis comes from mouse models with PLAUR overexpression, which exhibit increased vascular permeability and enhanced inflammatory signaling [38, 39].

Functional enrichment analysis validates the pathological significance of the identified genes. Disruptions in the organization of components within the aortic media, such as actin filament organization and other muscle system process GO terms, as well as disruptions in cell–substrate adhesion mechanisms, including cell–substrate adhesion and integrin binding, suggest critical effects of osNPy on cellular architecture and adhesion. Afterward, KEGG pathways enriched for PI3K‐Akt signaling, ECM‐receptor interaction, focal adhesion, TGF‐*β* signaling, and vascular smooth muscle contraction were previously implicated in the molecular mechanisms underlying vascular remodeling and dissection [17]. Significantly, the PI3K‐Akt pathway has been shown to regulate VSMC survival and proliferation, and pathological TGF‐*β* signaling is recognized in hereditary diseases affecting the aorta, including Marfan and Loeys–Dietz syndromes [17].

ITGA5 is a member of the integrin family and is crucial for the survival, differentiation, and proliferation of various cell types. It has been closely associated with multiple cancers, including cervical cancer, laryngeal squamous cell carcinoma, glioma, and gastric cancer [29–31]. Studies suggest that ITGA5 may interact with VEGFA through the AKT/VEGFA pathway, influencing tumor angiogenesis [31]. HGF acts on various cell types, including smooth muscle cells, fibroblasts, and endothelial cells, through its receptor cMet [32]. It regulates cell growth, motility, and morphogenesis and promotes tissue regeneration in damaged organs [33]. HGF activates and induces the proliferation and migration of endothelial cells, thereby influencing angiogenesis in multiple organs [32–35]. Additionally, HGF modulates the proliferation and differentiation of cardiomyocytes [35]. PLAUR, also known as uPAR, plays a pivotal role in tissue remodeling and ECM degradation, contributing to tumor cell invasion and metastasis [36]. It facilitates neuroprotection, cell proliferation, and angiogenesis [37]. Goodchild et al. demonstrated that mouse models with high PLAUR expression exhibit aortic tissues with a proinflammatory phenotype [38]. Additionally, Dergilev et al. confirmed in murine models that PLAUR plays a role in the formation and regulation of vascular wall components [39].

Although limited, our findings indicate that DPT is a promising biomarker for ATAAD. Second, the sample sizes in the microarray analysis and tissue validation were relatively small, reducing the robustness of the statistical conclusions. We attempted to mitigate this limitation by combining multiple datasets and validating them with unique samples. However, larger multicenter studies are needed. Second, gene discovery in microarray technology is limited to predefined probes, thereby missing novel transcripts and splice variants. Future work with RNA‐seq and single‐cell transcriptomics may reveal transcriptional heterogeneity in aortic tissues.

In addition, even though the functional assays show dramatic phenotype change after DPT knockdown, the full underlying signaling pathways (which dictate DPT functionality) are not fully determined yet. Whether DPT directly modulates these pathways or indirectly through interactions with integrins, TGF‐*β*, or other ECM proteins remains to be seen. Conditional in vivo models of ATAAD with DPT deletion in VSMCs could provide mechanistic insight and help determine whether it is a viable therapeutic target.

Finally, our global transcriptomic and experimental examination reveals that DPT is a downregulated regulator of HASMC behavior and a potential diagnostic and therapeutic biomarker for ATAAD. Simultaneous upregulation of ITGA5, HGF, and PLAUR indicates that an interplay between ECM remodeling, inflammation, and cell migration greatly complicates the pathogenesis of ATAAD. Further work is needed to translate these molecular discoveries into clinical interventions for this highly fatal disease.

## 5. Study Limitations

Despite the novel insights this study provides, several limitations should be acknowledged. First, our findings are based on relatively small sample sizes and publicly available microarray datasets, which may limit the statistical power and generalizability of our conclusions. Although we mitigated this by combining multiple cohorts and expanding the validation cohort, future studies with larger, multicenter datasets are needed. Second, the use of microarray technology limits transcriptomic discovery to predefined probes and fails to capture novel transcripts or cell‐type–specific expression. Future work using RNA sequencing and single‐cell transcriptomics could offer a more comprehensive view of gene regulation in ATAAD. Third, while our in vitro experiments demonstrate significant effects of DPT on VSMC migration and proliferation, the underlying signaling pathways and their causal roles in vivo remain to be elucidated. Conditional knockout mouse models and animal experiments are required to confirm the therapeutic relevance of DPT in disease progression. Finally, a critical limitation of our study is that validation of DPT expression was confined to aortic tissue specimens. We agree that assessing DPT levels in plasma, serum, or circulating extracellular vesicles would be critical for the development of noninvasive diagnostic assays. However, such analyses require extensive sample collection, standardized processing, and additional ethical approvals, which were beyond the scope of the current work. We plan to address this in future studies by evaluating circulating DPT as a biomarker in prospective patient cohorts.

## 6. Conclusions

The integration of two datasets identified four key ATAAD‐related genes. In functional enrichment analyses, the cell–matrix adhesion and cell growth, integrin‐binding protein, ECM structural components, PI3K‐Akt signaling, focal adhesion, ECM‐receptor interaction, and cGMP‐PKG signaling pathways were identified as potentially related to ATAAD onset and progression. DPT was also found to impact HASMC proliferative activity. These data may provide a foundation for future research focused on ATAAD.

## Ethics Statement

The study fully adhered to the principles outlined in the Declaration of Helsinki (2013 revision), ensuring the rights, safety, and well‐being of all participants. Ethical approval for the study has been obtained from the Ethics Committee of First Affiliated Hospital of Bengbu Medical College (2018039, Bengbu, China).

## Consent

A signed consent form was obtained from all participants; the participation was voluntary. At any time and/or for any reason, the participants can terminate my continued involvement in this study.

## Disclosure

All authors and participants agree that the study results will be published.

## Conflicts of Interest

The authors declare no conflicts of interest.

## Author Contributions

Ting Wei: methodology, software, formal analysis, investigation, resources, data curation, writing—original draft preparation, and visualization. Xiaopeng Yang: methodology, validation, formal analysis, investigation, resources, and data curation. Chao Shi: conceptualization, writing—review and editing, supervision, and project administration.

## Funding

No funding was received for this manuscript.

## Supporting Information

Additional supporting information can be found online in the Supporting Information section.

## Supporting information


**Supporting Information 1** Table S1. Differentially expressed genes (DEGs).

**Supporting Information 2** Table S2. PPI network data.

**Supporting Information 3** Figure S1. Western blot validation for siRNA knockdown efficiency at the protein level. Figure S2. Negative control (isotype IgG) staining for DPT, ITGA5, HGF, and PLAUR antibodies.

## Data Availability

The experimental data used to support this study’s findings are available from the corresponding author upon request.
