# Supplementary material for: Potential Diagnostic and Therapeutic Uses of DPT in Acute Type A Aortic Dissection
Source: Cardiovasc Ther. 2026 Feb 6;2026:8896404. doi: 10.1155/cdr/8896404 (PMC12880953; doi:10.1155/cdr/8896404)
Supplement: Supplementary file 3 — Supporting Information 3 Figure S1. Western blot validation for siRNA knockdown efficiency at the protein level. Figure S2. Negative control (isotype IgG) staining for DPT, ITGA5, HGF, and PLAUR antibodies. [file CDR-2026-8896404-s003.pdf]

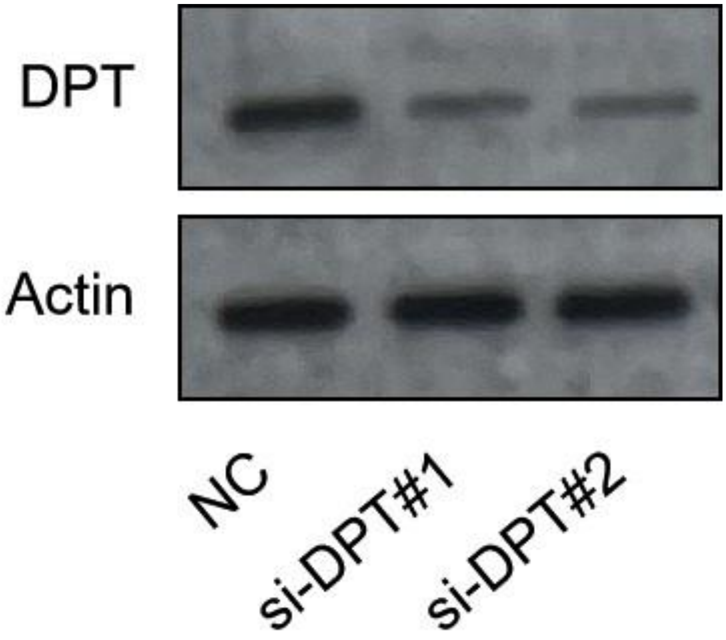

Figure S1. Western blot validation for siRNA knockdown efficiency at the protein level

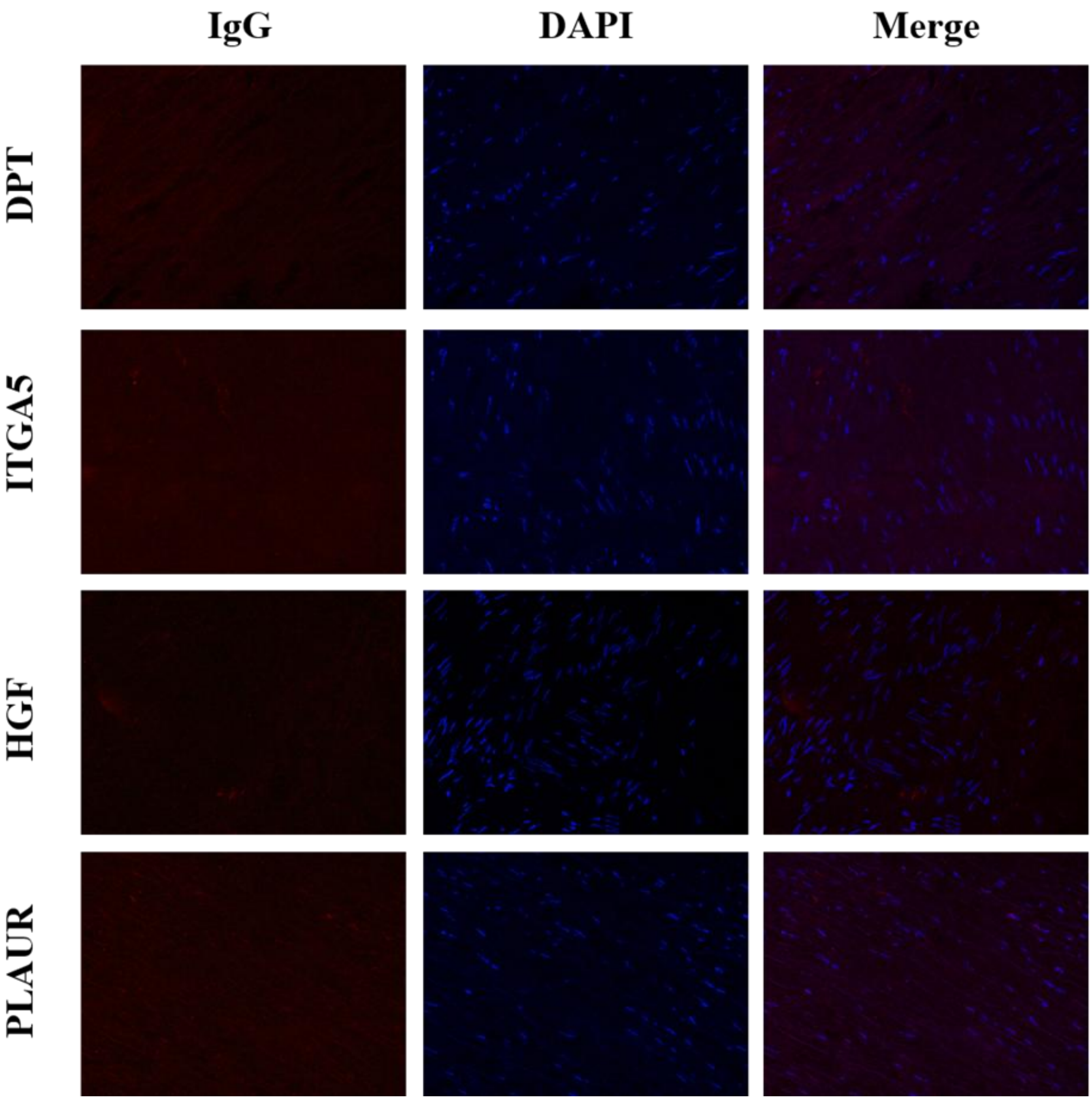

Figure S2: Negative control (isotype IgG) staining for DPT, ITGA5, HGF, and PLAUR antibodies.
